# Windei, the *Drosophila* Homolog of mAM/MCAF1, Is an Essential Cofactor of the H3K9 Methyl Transferase dSETDB1/Eggless in Germ Line Development

**DOI:** 10.1371/journal.pgen.1000644

**Published:** 2009-09-11

**Authors:** Carmen M. Koch, Mona Honemann-Capito, Diane Egger-Adam, Andreas Wodarz

**Affiliations:** 1 Abteilung Stammzellbiologie, DFG Research Center for Molecular Physiology of the Brain (CMPB), Georg-August-Universität Göttingen, Göttingen, Germany; 2 Fakultät für Biologie, Universität Konstanz, Konstanz, Germany; European Molecular Biology Laboratory, Germany

## Abstract

The epigenetic regulation of gene expression by the covalent modification of histones is a fundamental mechanism required for the proper differentiation of germ line cells during development. Trimethylation of histone 3 lysine 9 (H3K9me3) leads to chromatin silencing and the formation of heterochromatin by recruitment of heterochromatin protein 1 (HP1). dSETDB1/Eggless (Egg), the ortholog of the human methyltransferase SETDB1, is the only essential H3K9 methyltransferase in *Drosophila* and is required for H3K9 trimethylation in the female germ line. Here we show that Windei (Wde), the *Drosophila* homolog of mouse mAM and human MCAF1, is an essential cofactor of Egg required for its nuclear localization and function in female germ line cells. By deletion analysis combined with coimmunoprecipitation, we have identified the protein regions in Wde and Egg that are necessary and sufficient for the interaction between the two proteins. We furthermore identified a region of Egg that gets covalently modified by SUMOylation, which may facilitate the formation of higher order chromatin-modifying complexes. Together with Egg, Wde localizes to euchromatin, is enriched on chromosome 4, and binds to the Painting of fourth (POF) protein. Our data provide the first genetic and phenotypic analysis of a mAM/MCAF1 homolog in a model organism and demonstrate its essential function in the survival of germ line cells.

## Author Summary

Germ line cells are the only cells in an organism that are able to transmit their genetic material to the next generation by forming eggs or sperm. They do not participate in the formation or function of tissues and organs and therefore show a unique pattern of transcription, with many genes being silenced that are only required for somatic functions. The covalent modification of histones by methylation, acetylation, and other mechanisms is crucial for these global alterations in the transcriptional program. Among the modifications involved in silencing of chromatin regions, methylation of histone 3 lysine 9 (H3K9) is among the most important ones. Methylation of this residue in *Drosophila* is controlled by three different histone methyl transferases, but only one of these, dSETDB1/Eggless, is essential for viability and fertility of the fly. Here we describe an essential cofactor for dSETDB1/Eggless that is specifically required in germ line cells for their survival. This cofactor, that we called Windei, binds to dSETDB1/Eggless and recruits it to the nucleus. Null mutations in *windei* show strongly reduced trimethylation of H3K9 in germ line cells, demonstrating that Windei is one of the factors required for controlling chromatin organization in the germ line.

## Introduction

The epigenetic regulation of gene expression by the modification of histone proteins is a very important mechanism to control the differentiation of many cell types during development. The N-terminal, outward protruding histone tails are targets of posttranslational modifications, such as acetylation, ubiquitination, phosphorylation and methylation. These histone modifications are supposed to act sequentially or in combination to form a histone code that can be deciphered by different chromatin-associated proteins to mediate changes in chromatin structure and transcriptional activity [1]–[3].

One of the best-studied histone modifications is the methylation of the histone 3 lysine residue 9 (H3K9), which generally correlates with transcriptional repression [4]–[7]. However, recent results also point to a function of H3K9 methylation in the dynamic regulation of transcription, since this histone modification has frequently been found in the chromatin of actively transcribed genes [8]. H3K9 can be mono- di- or trimethylated and it has been shown that promoter H3K9 trimethylation results in much stronger transcriptional repression than promoter H3K9 dimethylation [9]. Methylated H3K9 can recruit Heterochromatin Protein 1 (HP1) [10]–[13], a chromatin-associated protein that has been implicated in heterochromatin formation but may also function in the regulation of euchromatic genes [14]. HP1 is highly conserved from yeast to human and was first found in *Drosophila* as a suppressor of position effect variegation Su(var)2–5 [15],[16].

Several histone methyltransferases (HMTs) have been identified that specifically methylate H3K9, the first being Su(var)3–9 of *Drosophila* [17], which is required for di- and trimethylation of H3K9 at the chromocenter [18]. Mammalian homologs of Su(var)3–9 are predominantly associated with constitutive heterochromatin [19],[20] and have been implicated in the regulation of telomere length [21]. G9a is a second H3K9 specific HMT which catalyzes mono- and dimethylation of H3K9 at euchromatic loci of mammalian cells [22]. G9a and its close relative GLP/Eu-HMTase1 form a heteromeric complex and appear to function cooperatively in the regulation of euchromatic genes [23].

A third class of H3K9 specific HMTs is represented by SETDB1/ESET [24],[25]. SETDB1 can be recruited to euchromatin by binding to KAP1/KRAB-ZFP transcriptional repressor complexes and functions in gene silencing by local methylation of H3K9 [24],[26]. In contrast to Su(var)3–9 and G9a HMTs, recombinant GST-SETDB1 fusion proteins have little HMT activity in vitro [24]. This is most likely caused by the requirement for binding to mAM/MCAF1, a protein copurifying with SETDB1 in mammalian nuclear extracts [9]. Knock-down of mAM by RNAi leads to an increase of H3K9me2, caused by the failure of SETDB1 to convert H3K9me2 to H3K9me3 [9]. mAM can bind simultaneously to SETDB1 and to the methyl CpG binding protein MBD1 and thus may provide a link between DNA methylation at CpG dinucleotides and histone H3K9 methylation mediated by SETDB1 [27],[28].

Knockout mice lacking the function of SETDB1 [29], Suv39h1 and Suv39h2 [30], G9a or its close relative GLP [23],[31] are all embryonic lethal, albeit at different developmental stages, demonstrating that these enzymes are essential and apparently have non-redundant functions.

Many proteins involved in transcriptional repression are either covalently modified by conjugation to the small ubiquitin-related modifier (SUMO) or they contain SUMO binding domains [32]. Binding to SUMO has been reported for both MCAF1 [33] and for SETDB1 [34]. It is generally thought that SUMOylation and binding to SUMO contributes to the efficient assembly of large protein complexes that allow the coordinated modification of multiple histone tail residues during the formation of heterochromatin.

In *Drosophila*, only the SETDB1 homolog dSETDB1/Eggless (Egg) is essential for viability and fertility [35]–[38], whereas mutants for Su(var)3–9 [17] and G9a [39] are homozygous viable and fertile. In polytene chromosome squash preparations, Egg localizes to euchromatic regions and is strongly enriched on chromosome 4 [35]. *egg* mutants loose most of the H3K9 methylation marks as well as binding of HP1 on chromosome 4, which is consistent with global changes in the transcription level of genes located on chromosome 4 that were observed in *egg* mutants [35],[37]. Egg coimmunoprecipitates with the chromosome 4 associated Painting of fourth (POF) protein [37], which is required for chromosome-wide transcriptional upregulation of genes on chromosome 4 [40],[41]. Homozygous *egg* mutant females possess only rudimentary ovaries, due to massive apoptosis at early stages of oogenesis in somatic and germ cells [36],[38]. H3K9me3 levels were strongly reduced in *egg* mutant germ line cells, particularly at the earliest stages of oogenesis in the germarium [36],[38].

So far it was not known whether Egg requires a binding partner homologous to mammalian mAM/MCAF1 for its function. Here we show that Windei (Wde), the *Drosophila* ortholog of mAM/MCAF1 precisely colocalizes with Egg in ovaries and binds to Egg in vivo. We furthermore show that Egg gets covalently modified by SUMOylation, which is a hallmark of many chromatin-associated proteins involved in transcriptional repression. Wde localizes to euchromatic regions of salivary gland polytene chromosomes, in particular to chromosome 4, and associates with POF in a protein complex. We have generated null mutations in *wde*, which are homozygous lethal and can be fully rescued by a transgene encoding a GFP-Wde fusion protein. Surviving homozygous *wde* mutant females are sterile and possess only rudimentary ovaries. Loss of *wde* function in germ line clones eliminates nuclear localization of Egg, leads to the arrest of oogenesis before stage 10 and to subsequent degeneration of mutant egg chambers by apoptosis. Like *egg* mutant cells, germ line cells mutant for *wde* show strongly reduced H3K9 trimethylation. According to the indistinguishable subcellular localization and mutant phenotypes of the two interactors, we propose that Wde is an essential binding partner of Egg required for the conversion of H3K9me2 to H3K9me3.

## Results

### CG12340 is the *Drosophila* homolog of mAM/MCAF1

**Figure 1:**
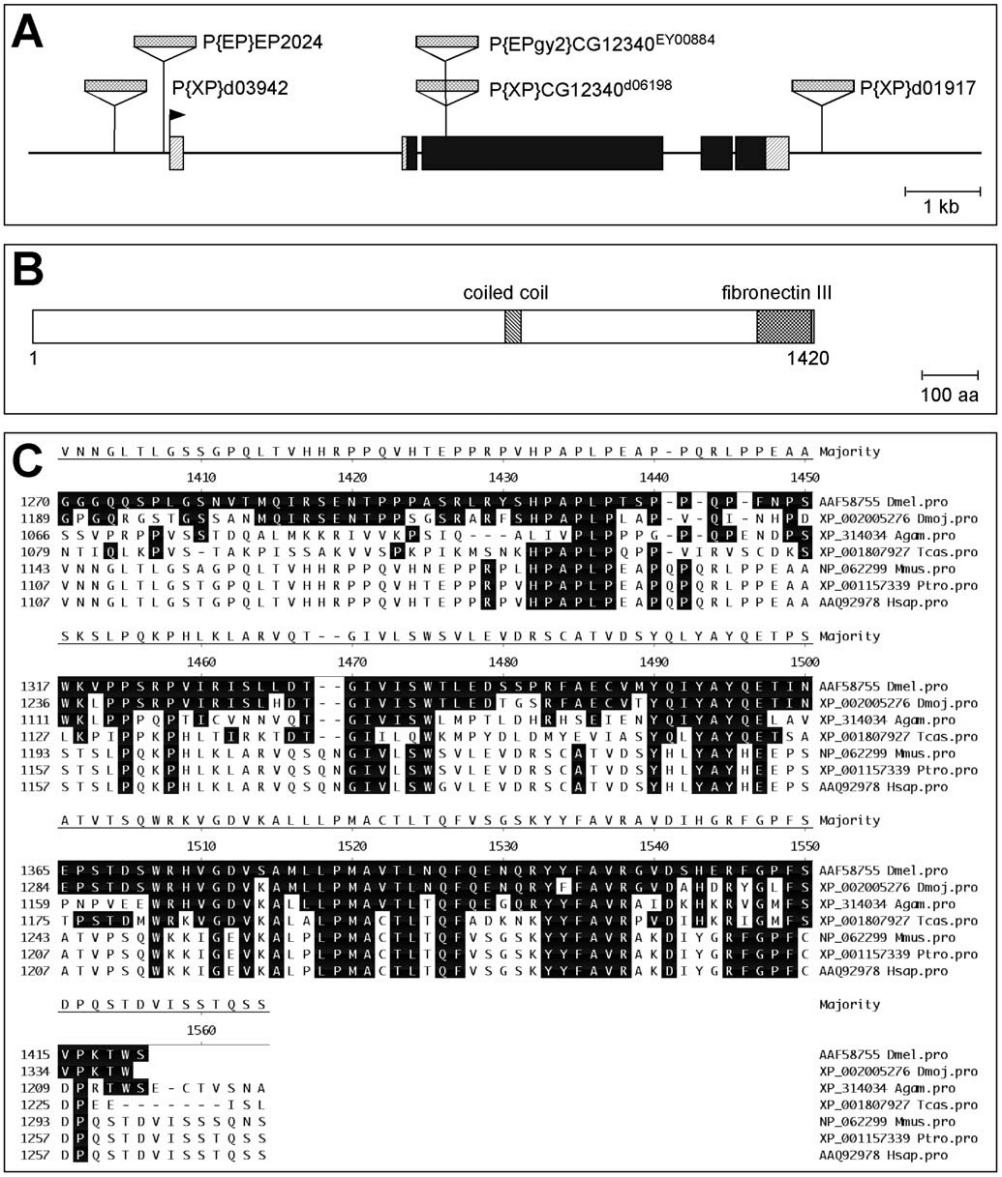
Genomic structure of the *wde* locus and structure of the Wde protein. (A) The *wde* locus comprises 5 exons and the longest ORF starts in the second exon. P-element insertions relevant for this study are indicated (P elements are not drawn to scale). The transcription start site is marked by a flag. 5′ and 3′ UTRs are hatched and the ORF is black. (B) The predicted Wde protein is 1420 amino acids long and contains a coiled-coil region in the center and a C-terminal fibronectin type III domain. Domain predictions were made using the SMART algorithm (http://smart.embl-heidelberg.de/). (C) Alignment of the C-terminal region of Wde including the fibronectin type III domain (aa 1320–1411) with the closest homologs of Wde from *Drosophila mojavensis* (Dmoj), the moscito *Anopheles gambiae* (Agam), the red flour beetle *Tribolium castaneum* (Tcas), the mouse *Mus musculus* (Mmus), the chimp *Pan troglodytes* (Ptro), and human (Hsap). The alignment was made with Megalign (DNAStar Inc.) software. Sequence names correspond to the GenBank accession numbers. Residues identical to the Wde sequence are boxed in black.

In human cells, conversion of dimethyl to trimethyl H3K9 by the histone methyl transferase SETDB1/ESET is greatly facilitated by binding of this enzyme to mAM/MCAF1 (also called ATFa associated factor) [9]. The *Drosophila* homolog of SETDB1/ESET called dSETDB1/Eggless (Egg) is essential for oogenesis [36],[38] and for H3K9 trimethylation on chromosome 4 [35],[37]. So far it was not known whether Egg activity requires a cofactor homologous to mAM. Database screening using the BLAST algorithm (http://blast.ncbi.nlm.nih.gov/Blast.cgi) revealed the existence of a single *Drosophila* homolog of mAM encoded by the *CG12340* transcription unit located at position 47C1 on the right arm of the second chromosome (Figure 1A). Due to its mutant phenotype (see below) we named this gene *windei* (*wde*, german for wind egg) and will use this name throughout the manuscript. *wde* encodes a strongly acidic protein (pI = 4,55) of 1420 amino acids and a calculated molecular weight of 157.776 Dalton. Although the overall sequence identity between mAM and Wde is only 14,8%, the domain structure with an internal coiled-coil region and a C-terminal fibronectin type III repeat is identical (Figure 1B). Within the fibronectin type III repeat, the sequence identity is 36% (57% similarity) (Figure 1C).

### Wde is a ubiquitously expressed nuclear protein that colocalizes with Egg in the female germ line

In order to study the expression pattern and subcellular localization of Wde, we raised specific antibodies against two peptides corresponding to aa 70–84 and aa 1286–1301. The specificity of the antibodies was tested in stainings of wild type, *wde* mutant and Wde overexpressing embryos (Figure S1) and in ovaries containing *wde* mutant germ line clones (Figure S3). For all analyses shown here, we used antiserum affinity purified against the peptide coprresponding to aa 70–84.

**Figure 2:**
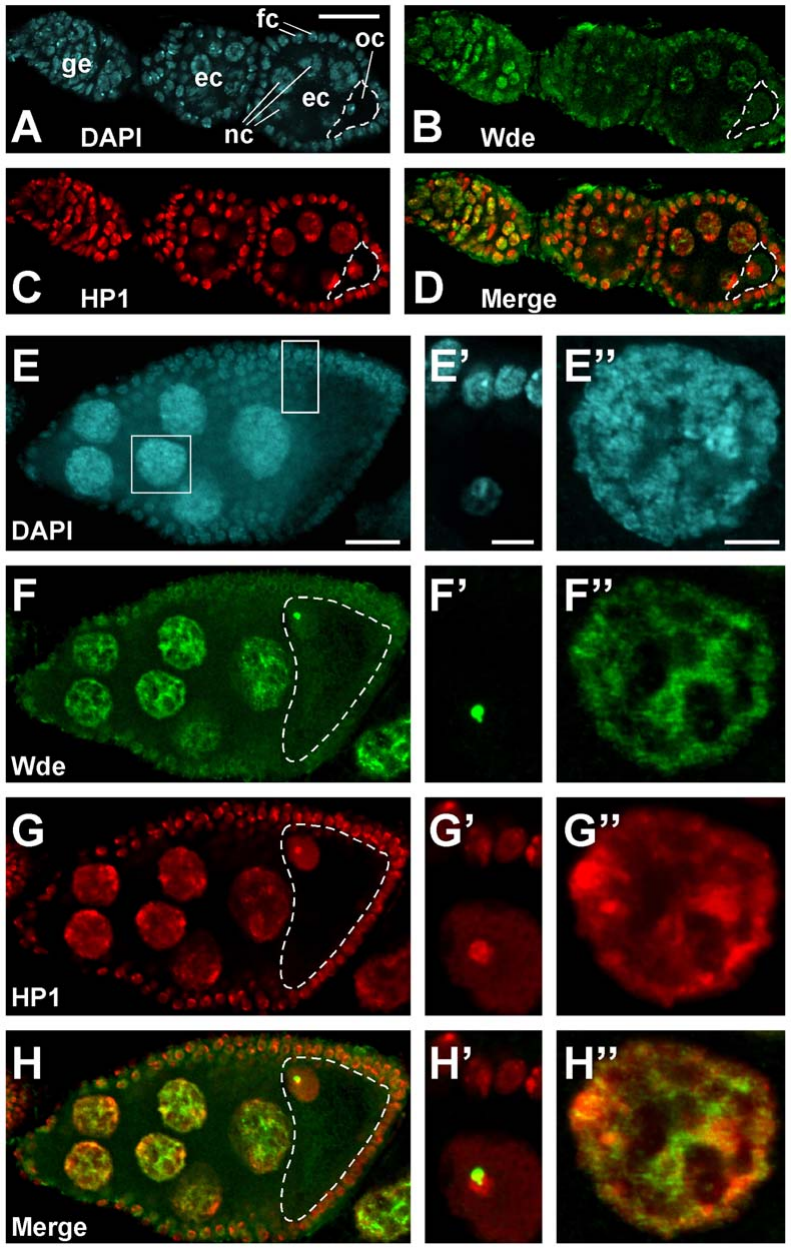
Localization of Wde during oogenesis. (A–D) Wde is expressed both in somatic cells and in germ line cells of ovaries during early stages of oogenesis. Shown is the tip of an ovariole containing the germarium (ge) and two egg chambers (ec) stained for DNA (DAPI, (A)), Wde (B), and heterochromatin protein 1 (HP1, (C)). The merged image of Wde and HP1 is shown in (D). fc, follicle cell; nc, nurse cell; oc, oocyte. (E–H) Wde and HP1 are localized to subnuclear structures and are only partially colocalized. An egg chamber at stage 8 was stained for DNA (DAPI, (E)), Wde (F), and HP1 (G). The merged image of Wde and HP1 is shown in (H). Insets corresponding to the boxes in (E) are shown to the right of the respective images (E'–H''). Scale bars in (A) and (E) = 20 µm. Scale bars in the insets (E') and (E'') = 5 µm. Oocytes (oc) are outlined with a white dotted line. Anterior is to the left in all images.

Because mutant flies deficient for Egg, the potential binding partner of Wde, show severe defects during oogenesis [36],[38], we focused our analysis on the subcellular localization of Wde in ovaries. Wde was ubiquitously expressed both in the somatic follicle cells and in germ line cells at all stages of oogenesis (Figure 2). Wde was nuclear in interphase (Figure 2B and 2F) and localized in the cytoplasm in mitosis after nuclear envelope breakdown (Figure S1K, Figure S1L, Figure S1M). Within the nucleus, Wde was not homogeneously distributed but showed a reproducible localization to subnuclear structures (Figure 2F). This was particularly obvious in the oocyte nucleus and in the highly polyploid nurse cell nuclei (Figure 2F and 2F'''). To determine more precisely to which structure Wde localized in the nucleus, we performed double stainings with an antibody against HP1. HP1 is enriched in heterochromatin, in particular at the chromocenter, the centromeric heterochromatin in which all four chromosomes of *Drosophila* are attached to each other during interphase [42]. In the oocyte nucleus, Wde always was present in one or 2 very brightly staining dots that were in close apposition, but not colocalizing with the brightest spot of HP1 staining at the chromocenter (Figure 2F–2H'). In nurse cell nuclei, Wde and HP1 colocalized to some extent, but there were also regions where only one of the two proteins was detectable (Figure 2F–2H'').

**Figure 3:**
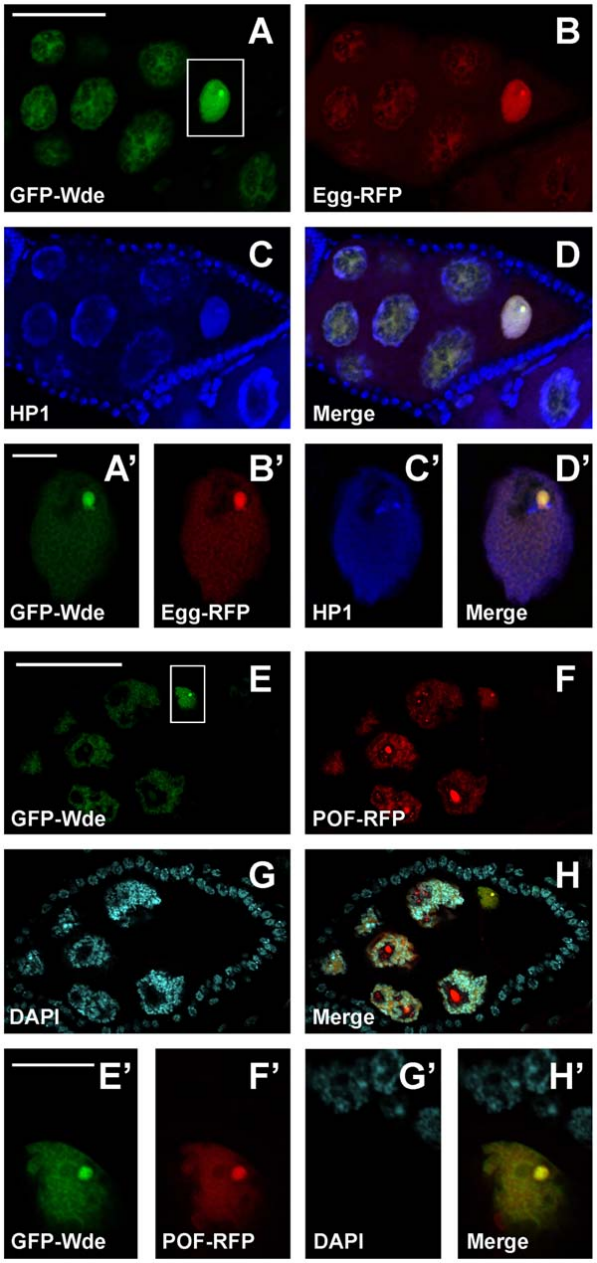
GFP-Wde, Egg-RFP, and POF colocalize in the oocyte nucleus. (A–D) GFP-Wde (A) and Egg-RFP (B) were coexpressed in the female germ line using the *daughterless*-GAL4 driver line. Egg chambers were also stained for HP1 (blue; (C)). (E–H) GFP-Wde (E) and POF-RFP were coexpressed in the female germ line using the *daughterless*-GAL4 driver line. Note that the large blobs of POF-RFP in nurse cell nuclei that do not colocalize with GFP-Wde are aggregation artefacts caused by overexpression. (A'–D') and (E'–H') show higher magnifications of the area surrounding the oocyte nucleus boxed in (A) and (E), respectively. Scale bars in (A,E) = 50 µm, scale bars in (A',E') = 10 µm. Anterior is to the left.

The published localization pattern of Egg [36] was strikingly similar to that of Wde. In order to test whether the two proteins indeed colocalize, we generated transgenic flies expressing a full length Egg-RFP fusion protein, which resembled precisely the published localization pattern of Egg. Double stainings of endogenous Wde with Egg-RFP (data not shown) and of GFP-Wde with Egg-RFP revealed that both proteins colocalized exactly (Figure 3). This was also true for the prominent dots in the oocyte nucleus (Figure 3A'–3D'). To test whether Wde also colocalized with POF, a known binding partner of Egg [35],[37], we coexpressed GFP-Wde and POF-RFP in germ line cells (Figure 3E–3H). Both proteins colocalized precisely in the oocyte nucleus, showing that the prominent dot that stained for Wde, Egg and POF corresponds to the fourth chromosome.

### Wde associates with Egg in a protein complex

**Figure 4:**
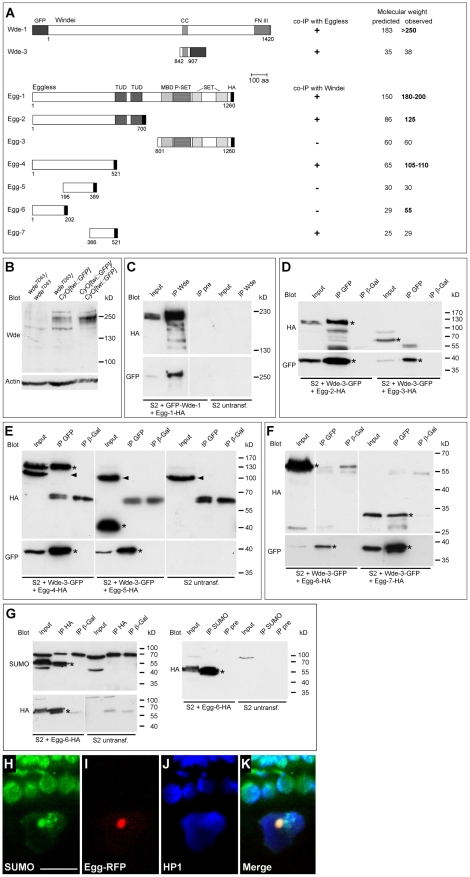
Wde and Egg are associated in a protein complex. (A) A series of constructs encoding epitope-tagged full-length and deletion versions of both Wde and Egg was generated for expression in S2 cells. Protein domains identified by the SMART algorithm are indicated by boxes with different types of hatching. Abbreviations: CC, coiled coil; FN III, fibronectin type III; GFP, green fluorescent protein; TUD, Tudor; MBD, Methyl-CpG binding domain; P-SET, preSET; SET, Su(var)3–9, Enhancer-of-zeste, Trithorax; HA, hemagglutinin epitope tag. GFP and HA epitope tags are not drawn to scale. Numbers indicate the region of the protein that is present in the respective construct (in aa). Coimmunoprecipitation of the respective proteins with Egg and Wde and the predicted and observed molecular weight of the tagged proteins are indicated to the right. (B) Protein extracts of homozygous *wde^TD63^* null mutant embryos, embryos heterozygous for the *wde^TD63^* null mutant and embryos homozygous for the balancer chromosome (wild type for *wde*) were separated by SDS-PAGE and blotted with the anti Wde antiserum. An antibody against actin was used as loading control. (C) GFP-Wde-1 and Egg-1-HA (see Figure 4A) were cotransfected into S2 cells. Immunoprecipitates were collected using the anti Wde antiserum (IP Wde) or the corresponding preimmune serum as negative control (IP pre). Untransfected S2 cells were used as additional control. Immunoprecipitates were blotted with antibodies against HA (top) and GFP (bottom). (D) S2 cells were cotransfected with Wde-3-GFP and either Egg-2-HA or Egg-3-HA. Immunoprecipitates were collected with either anti GFP antibody (IP GFP) or with an antibody against β-Galactosidase (IP β-Gal) as negative control. (E) S2 cells were cotransfected with Wde-3-GFP and either Egg-4-HA or Egg-5-HA. (F) S2 cells were cotransfected with Wde-3-GFP and either Egg-6-HA or Egg-7-HA. In (D–F), the bands corresponding to the expressed GFP- and HA-tagged fusion proteins are marked by asterisks. Arrowheads in (E) mark an unspecific band that is detected by the anti-HA antibody in S2 cell lysates. (G–K) Egg is covalently modified by SUMOylation. (G) An HA-tagged N-terminal fragment of Egg (Egg-6-HA, aa 1–202) was expressed in S2 cells, immunoprecipitated with anti-HA antibody and blotted against SUMO (top left) and HA (bottom left). In the reciprocal experiment, immunoprecipitation was done with anti SUMO, followed by Western blot against HA (right). Immunoprecipitation with anti β-galactosidase (β-Gal) and untransfected S2 cells served as negative controls. Relevant bands are marked by asterisks. (H–K) SUMO colocalizes with Egg-RFP in the oocyte nucleus. Ovaries in which Egg-RFP (I) was expressed under control of *daughterless*-GAL4 were stained for SUMO (H) and HP1 (J). Scale bar = 10 µm.

The precise colocalization of Wde with Egg and the fact that the mammalian homologs of Wde and Egg bind to each other [9] prompted us to test whether Wde and Egg associate in a protein complex. To that aim, we generated a series of full length and partially deleted GFP-Wde and Egg-HA fusion proteins (Figure 4A and Text S1) for expression in *Drosophila* S2 cells. To test our anti Wde antibody for specificity in Western blots, we used extracts from wild type embryos and from embryos homozygous mutant for a null allele of *wde* (see below). In wild type embryos, the antibody detected several bands with a molecular weight around 250 kD that were absent in extracts of homozygous mutant embryos (Figure 4B). We then coexpressed full length GFP-Wde with full length Egg-HA. Coimmunoprecipitation with the anti Wde antibody, followed by Western blot with antibodies against GFP and HA showed that the antibody precipitated GFP-Wde and Egg-HA, demonstrating that both proteins were associated in a complex (Figure 4C). The same result was obtained when anti-GFP antibody was used instead of the affinity-purified antiserum against Wde (data not shown). To narrow down the regions of both proteins that were required for complex formation, we coexpressed different deletion constructs for both proteins (Figure 4A) and tested them by coimmunoprecipitation. These experiments revealed that a fragment of Wde containing the coiled-coil-region (aa 842–907) was sufficient for coimmunoprecipitation of Egg (Figure 4D–4F). The smallest fragment of Egg required for coimmunoprecipitation with Wde consisted of aa 366–521 (Figure 4A, 4D–4F), a region that does not contain any known protein motif detected by the SMART algorithm (http://smart.embl-heidelberg.de/).

### Egg is modified by SUMOylation

Many proteins involved in transcriptional repression can either bind to SUMO and SUMOylated proteins or are covalently modified by SUMOylation. We noticed that full length Egg ran at a higher molecular weight in SDS-PAGE than predicted from its sequence (Figure 4A). This was also true for all fragments of Egg that contained the N-terminal 202 aa (Figure 4A), suggesting that this region is covalently modified. To test whether aa 1–202 of Egg are SUMOylated, we expressed this part of Egg fused to HA (Egg-6-HA; Figure 4A) in S2 cells, immunoprecipitated the protein with HA antibody and probed the Western blot with an antibody against SUMO (Figure 4G). The immunoprecipitated 55 kD band corresponding to Egg-6-HA was clearly recognized by the SUMO antibody (Figure 4G). In the reverse experiment, Egg-6-HA was detected in immunoprecipitates pulled down with the SUMO antibody (Figure 4G), confirming that Egg-6-HA was modified by SUMOylation. To address the in vivo relevance of these observations, we stained egg chambers expressing Egg-RFP with antibodies against SUMO and HP1 (Figure 4H–4K). Consistent with our tissue culture data, SUMO colocalized with the dot of Egg-RFP on the fourth chromosome in the oocyte nucleus (Figure 4H–4K).

### Wde localizes to euchromatic regions of salivary gland polytene chromosomes, is enriched on chromosome 4, and binds to POF

**Figure 5:**
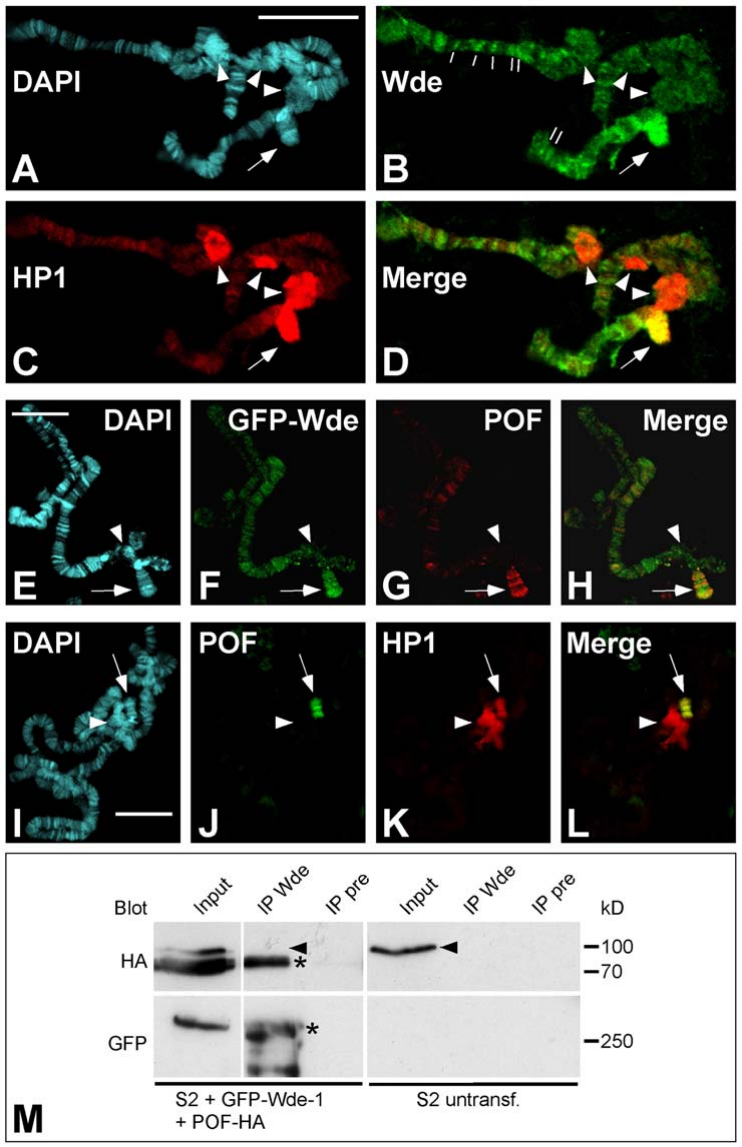
Wde localizes to euchromatic regions of salivary gland polytene chromosomes, is enriched on chromosome 4, and binds to POF. (A–D) Squash preparations of wild type salivary gland polytene chromosomes were stained for DNA with DAPI (A) and with antibodies against Wde (B) and HP1 (C). (B) Wde staining is most prominent on chromosome 4 (arrow) and in several bands along the euchromatic arms of the chromosomes (lines). No prominent staining of Wde is detectable on the chromocenter (arrowheads). (C) HP1 is also present on chromosome 4 (arrow) and shows intense staining of the chromocenter (arrowheads). Note that the chromocenter in this squash preparation has been ruptured and appears as three distinct spots. (E–H) Salivary gland polytene chromosome preparations of larvae overexpressing GFP-Wde under control of *daughterless*-GAL4 were stained for DNA (DAPI; (E)), GFP (F), and POF (G). (I–L) Squash preparations of wild type salivary gland polytene chromosomes were stained for DNA with DAPI (I) and with antibodies against POF (J) and HP1 (K). Scale bars = 20 µm. (M) GFP-Wde-1 and POF-HA were cotransfected into S2 cells. Immunoprecipitates were collected using the anti Wde antiserum (IP Wde) or the corresponding preimmune serum as negative control (IP pre). Untransfected S2 cells were used as additional control. Immunoprecipitates were blotted with antibodies against HA (top) and GFP (bottom). Bands corresponding to the expressed GFP- and HA-tagged fusion proteins are marked by asterisks. Arrowheads mark an unspecific band that is detected by the anti-HA antibody in S2 cell lysates.

To find out whether Wde is a chromatin-associated protein, we performed immunofluorescence stainings on squashed salivary gland polytene chromosomes. Wde was not present in significant amounts on the chromocenter, but intense staining was detectable on the fourth chromosome that was also stained by the HP1 antibody (Figure 5B–5D). In addition, Wde was present in several bands in the euchromatic region of all chromosomes (Figure 5B). The enrichment of Wde on the fourth chromosome was confirmed by double stainings of GFP-Wde and Painting of fourth (POF), a protein that binds predominantly to the fourth chromosome of *Drosophila melanogaster* (Figure 5F–5H) [41]. POF staining overlapped with HP1 only on the fourth chromosome, but not on the chromocenter (Figure 5J–5L). To test whether Wde and POF were associated with each other in a protein complex, we coexpressed full-length GFP-Wde and full length POF-HA in S2 cells. Upon coimmunoprecipitation using an antibody against GFP, both GFP-Wde and POF-HA were detectable in Western blots (Figure 5M), demonstrating that both proteins were present in one complex.

### *wde* is an essential gene

To analyze the function of *wde* in development, we generated a null mutation of *wde* (*wde^TD63^*) by FLP/FRT mediated recombination in trans of two P-elements flanking the *wde* locus on both sides (Figure 1A; for details see Materials and Methods). Two additional mutant alleles (*wde^00884^* and *wde^06198^*) caused by insertion of the P-elements P{Epgy2}CG12340^EY00884^ and P{XP}CG12340^d06198^, respectively, into the coding region of *wde* (Figure 1A) are predicted to result in premature termination of translation and are likely to be null alleles as well. Animals homozygous for any of the three mutant alleles or transheterozygous for any combination of the three alleles die at pupal stages. However, rare escapers were obtained by raising homozygous mutant larvae separated from their heterozygous siblings (see Materials and Methods), which eliminates competition for food and allows the weak mutants to reach the adult stage. Adult homozygous mutant animals were very weak and survived only for few days. The ovaries of homozygous *wde* mutant females were tiny and did not develop to the stage when egg chambers bud off from the germarium (Figure S2). The lethality and the ovary phenotype of the *wde^TD63^* null allele was fully rescued by ubiquitous expression of the full length GFP-Wde fusion protein under control of *daughterless*::GAL4 using the UAS/GAL4 system (data not shown), proving that the observed defects were due to mutation of *wde* and not to a second site mutation elsewhere in the genome.

### Wde is required in germ line cells for survival and for trimethylation of H3K9

**Figure 6:**
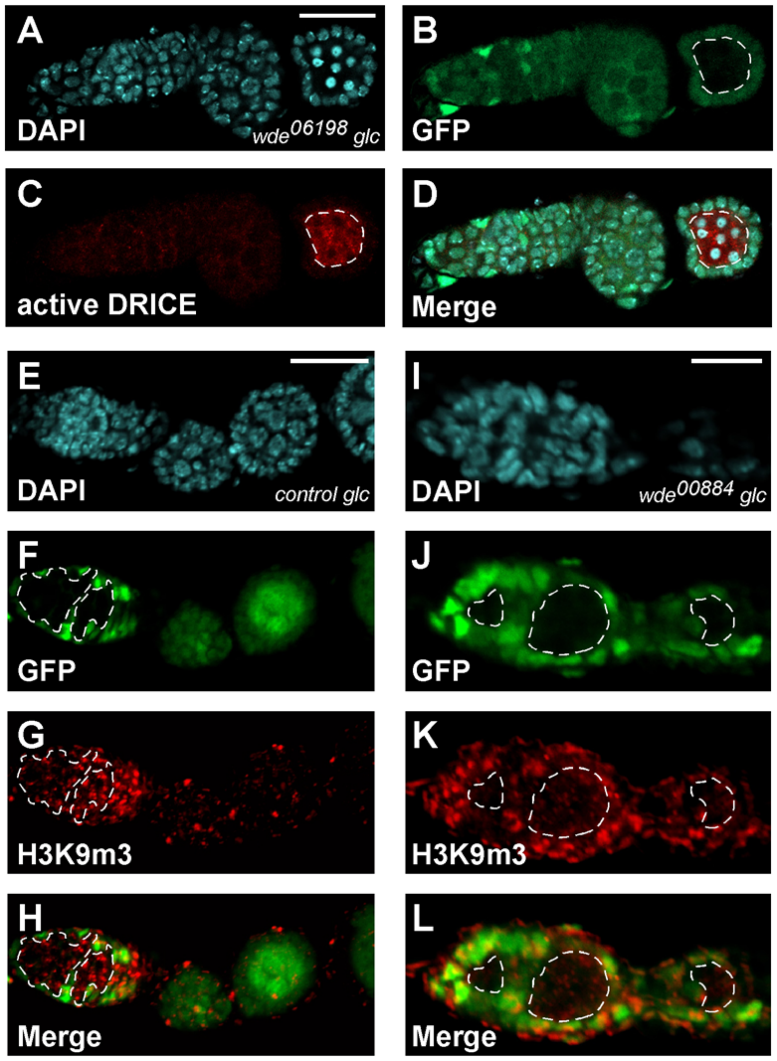
Loss of *wde* function in the germ line leads to apoptosis and to reduced trimethylation of H3K9. (A–D) *wde^06198^* mutant germ line cells enter apoptosis at later stages of egg chamber development. Clones are marked by the absence of GFP fluorescence ((B), dotted white circle). Apoptosis was detected by condensation of chromatin visible by brighter DAPI staining (A) and by strongly increased staining for the activated caspase Drice (C). (E–L) Wde is required for trimethylation of H3K9 in germ line cells. Ovaries containing GFP marked control germ line clones (E–H) and *wde^00884^* mutant germ line clones (I–L) were stained for DNA with DAPI (E,I) and for H3K9me3 (G,K) using a specific antibody. Scale bars = 20 µm. Anterior is to the left.

To analyze the requirement for *wde* during germ line development without affecting the function of *wde* in follicle cells, we generated germ line clones of the *wde^00884^* and *wde^TD63^* alleles using the autosomal FLP-DFS technique [43]. *wde^00884^* and *wde^TD63^* germ line clones did not produce any eggs, whereas control clones using the same FRT chromosome without a *wde* mutation produced eggs at the expected frequency (data not shown). To analyze the oogenesis defect of *wde* mutants in more detail, we generated germ line clones marked by the absence of GFP expression using the FLP/FRT technique. While control clones showed robust nuclear Wde staining (Figure S3C and Figure S3E), clones mutant for *wde^00884^* completely lacked nuclear Wde staining (Figure S3H and Figure S3J). This was also true for the alleles *wde^06198^* and *wde^TD63^* (data not shown). We noted that egg chambers with *wde* mutant germ line clones did not develop beyond stage 8 of oogenesis. Closer inspection of late stage egg chambers with *wde* germ line clones revealed that the nuclei of germ line cells were highly condensed and degenerated subsequently (Figure 6A and 6D). This morphological feature is typical of cells undergoing apoptosis. Stainings of *wde* mutant ovaries with an antibody against the activated caspase Drice, a marker for apoptotic cells [44], showed a strong increase of Drice staining in *wde* mutant germ line cells (Figure 6C and 6D), demonstrating that *wde* is required for survival of germ line cells.

Previous work on the function of Egg during oogenesis had shown that this enzyme is required for trimethylation of H3K9, especially in the germarium [36],[38]. To check whether *wde* mutant germ line cells also show reduced levels of H3K9me3, we stained ovaries containing germ line clones of *wde* with an antibody against H3K9me3. Whereas H3K9me3 levels were unaffected in germaria with control germ line clones (Figure 6G and 6H), H3K9me3 staining was strongly reduced in clones of *wde* mutant germ line cells (Figure 6K and 6L).

### The phenotypes of *wde* and *egg* mutants are indistinguishable

The oogenesis phenotype of *egg* mutants has so far been only described for the rudimentary ovaries of homozygous mutant females [36],[38]. To compare the mutant phenotypes of *egg* and *wde* mutants, we generated germ line clones for the *egg^1473^* allele, which has a deletion of the SET domain and thus is nonfunctional with respect to its histone methyl transferase activity [36]. The reduction in H3K9me3 staining in *egg^1473^* germ line clones was as strong as in *wde* mutant germ line clones (data not shown), demonstrating that Wde is indispensable for H3K9 trimethylation by Egg. In general, the germ line clone phenotypes of *wde* and *egg^1473^* mutants were indistinguishable with respect to apoptosis and the timing and severity of egg chamber degeneration (data not shown). Moreover, germ line clones doubly mutant for *wde^06198^* and *egg^1473^* showed the same phenotype as either single mutant (data not shown), indicating that both proteins function in the same process and are nonfunctional in the absence of their binding partner.

### Wde is required for nuclear localization of Egg

**Figure 7:**
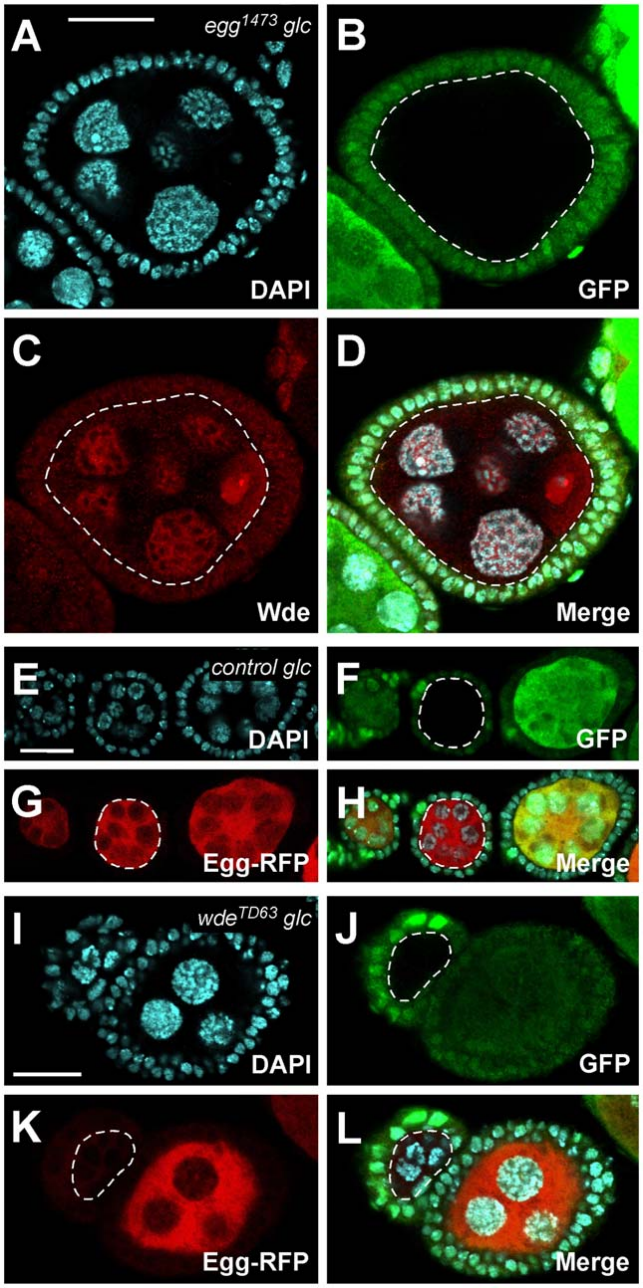
Wde is required for stabilization of Egg in germ line cells. (A–D) Ovaries with germ line clones of *egg^1473^* were stained for DAPI (A), GFP (B), and Wde (C). Germ line clones are marked by loss of GFP ((B–D), dotted white circles). (E–H) Control germ line clones and (I–L) germ line clones of the *wde^TD63^* null allele expressing Egg-RFP (G,K) were stained for DAPI (E,I) and GFP (F,J). Note that Egg-RFP levels in the cytoplasm are higher than in Figure 3 due to the heat shock applied for induction of germ line clones. Scale bars = 20 µm. Anterior is to the left.

To test whether Wde and Egg are dependent on each other for proper nuclear localization in germ line cells, we analyzed germ line clone ovaries with respect to the localization of both proteins. Wde was normally localized in germ line clones for *egg^1473^* (Figure 7C and 7D), whereas Egg-RFP was hardly detectable in germ line clones of the null allele *wde^TD63^* (Figure 7K and 7L), in contrast to control germ line clones with the same FRT chromosome carrying a wild type copy of *wde* (Figure 7G and 7H). These data clearly show that Wde is required for stabilization of Egg in germ line cells, but since *egg^1473^* is not a protein null allele they leave open the question of whether Egg is also required for proper localization of Wde.

**Figure 8:**
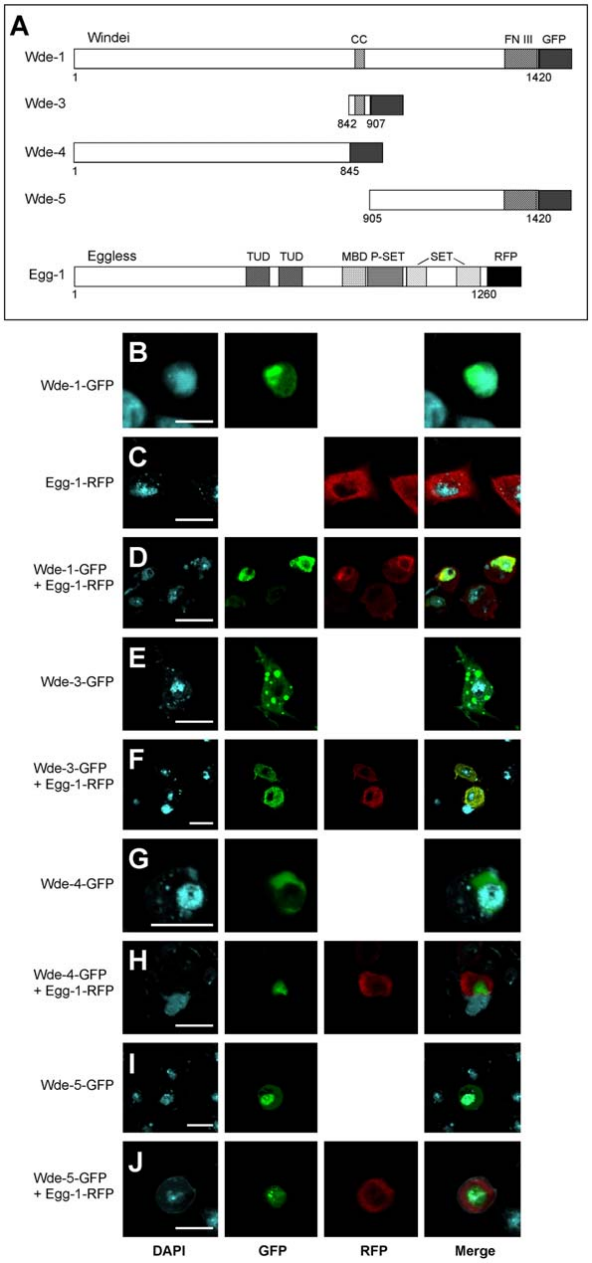
Wde is required for nuclear localization of Egg in S2 cells. (A) Constructs generated for transfection in S2 cells. (B) Wde-1-GFP localizes to the nucleus. (C) Egg-1-RFP localizes to the cytoplasm. (D) When coexpressed, Wde-1-GFP and Egg-1-RFP colocalize in the nucleus. (E) Wde-3-GFP localizes to the cytoplasm. (F) When coexpressed, Wde-3-GFP and Egg-1-RFP colocalize in the cytoplasm. (G) Wde-4-GFP localizes to a subcompartment in the cytoplasm. (H) When coexpressed, Wde-4-GFP and Egg-1-RFP localize to the cytoplasm but are not colocalized. (I) Wde-5-GFP localizes to the nucleus. (J) When coexpressed, Wde-5-GFP localizes to the nucleus and does not colocalize with Egg-1-RFP in the cytoplasm. Scale bars = 10 µm.

To clarify this issue, we analyzed the subcellular localization of GFP-tagged Wde and RFP-tagged Egg in transfected S2 cells. When transfected alone, Wde localized to the nucleus (Figure 8B) and Egg to the cytoplasm (Figure 8C). Cotransfection of full length Wde and Egg resulted in nuclear colocalization (Figure 8D). A deletion analysis of Wde (Figure 8A) revealed that the C-terminal region of Wde is required and sufficient for its nuclear localization (Figure 8G and 8I), and that the coiled-coil region is additionally required to recruit Egg to the nucleus (Figure 8J). From these results we conclude that Wde can localize to the nucleus in the absence of Egg and that Wde is required for nuclear localization of Egg.

## Discussion

### Wde is strictly required for the in vivo function of Egg

In this study, we have analyzed the function of Wde, the *Drosophila* homolog of mAM/MCAF1, in development. Wde precisely colocalizes with Egg and the mutant phenotypes of *wde* and *egg* are indistinguishable, indicating that Wde is an indispensable binding partner of Egg required for trimethylation of H3K9 at euchromatic sites, in particular on the fourth chromosome. Functional data on mAM/MCAF1 have so far only been obtained by RNAi-mediated knock-down [9], or by expression of mutated mAM/MCAF1 proteins in tissue culture cells [27]. The first study concluded that mAM/MCAF1 increases the enzymatic HMT activity of SETDB1, in particular with respect to the conversion of H3K9me2 to H3K9me3 [9]. The second study showed that expression of mAM/MCAF1 mutated in its binding site for MBD1 interferes with recruitment of SETDB1 to chromatin [27]. Our study is the first using a null mutant of a mAM/MCAF1 homolog und our results clearly show the strict requirement for Wde for proper localization and in vivo function of Egg in germ line cells.

### Wde is required for stability and nuclear localization of Egg

It was shown before that mAM/MCAF1 and SETDB1 associate in a protein complex and that a short region of mAM/MCAF1 including the coiled-coil domain is sufficient for binding to SETDB1 [9],[27]. We have confirmed and extended these observations by showing that a region including the coiled-coil domain of Wde is sufficient for binding to Egg and that a short region of Egg (aa 366–521) devoid of any known protein motif is sufficient for binding to Wde. For mAM/MCAF1 it was proposed that its binding to SETDB1 alters the catalytic activity and substrate specificity of the histone methyl transferase domain, thus allowing efficient trimethylation of H3K9 [9]. While the same may be true for the Wde/Egg interaction, our results show that in the absence of Wde, Egg is hardly detectable in germ line cells, most likely because Wde is required to protect Egg from proteolytic degradation. Moreover, when Egg is overexpressed in the absence of Wde, it fails to localize to the nucleus, revealing an additional function for Wde in nuclear import of Egg.

### Wde binds to POF and may control the transcription of genes located on chromosome 4

On polytene chromosomes Wde binds strongly to the fourth chromosome and to multiple euchromatic bands on all other chromosomes. Strong binding to the fourth chromosome has also been reported for Egg [35] and is consistent with the hypothesis that Egg may be specifically required for euchromatic H3K9 trimethylation on the fourth chromosome, which is not affected in *Su(var)3–9* and *G9a* mutants [18],[39]. Two recent studies showed indeed that Egg specifically affects the transcription of loci located on chromosome 4 [35],[37]. However, the two studies come to apparently contradictory results. While the first study [35] reported derepression of transgenes inserted on chromosome 4 in *egg* mutants, the second study [37] reported a general reduction of the transcription of genes on the fourth chromosome in *egg* mutants, measured in a microarray experiment. Nonetheless, the involvement of both Egg and Wde in the transcriptional regulation of genes on chromosome 4 appears very likely, since both Egg [37] and Wde (this study) bind to POF. We could not determine whether Wde and Egg bind to POF independently or sequentially, because we cannot exclude that the expression of endogenous Egg in S2 cells contributes to the binding of transfected Wde and POF.

POF is a unique example of a protein that specifically associates with a single autosome, the fourth chromosome of *Drosophila melanogaster* [41],[45]. In *pof* mutants, the transcription level of genes on the fourth chromosome is reduced, indicating that POF promotes transcription of genes on chromosome 4 [40]. On the other hand, the localization of POF to chromosome 4 is dependent on HP1 and vice versa, and there appears to be competition between these two proteins for binding to genes and their promoters on chromosome 4 [40],[46]. These observations have led to the model that the activities of HP1 and POF have to be balanced in order to ensure transcription of genes on chromosome 4 at the right level [40],[46]. We propose that Egg and Wde are part of this balancing mechanism because both proteins bind to POF and recruit HP1 by generating H3K9me3 marks on chromosome 4.

### A specific requirement for the Wde/Egg complex in development of the female germ line

Two recent studies showed that Egg is required for the development of ovaries in *Drosophila*. Ovaries of homozygous *egg* mutant females are rudimentary and degenerate by apoptosis before egg chambers bud off the germarium [36],[38]. We have confirmed this result and have shown that homozygous *wde* mutant females show exactly the same phenotype. From these observations it was not clear whether the function of Egg and Wde is required in the germ line cells, the somatic follicle cells, or both. To address this question, we eliminated the function of *egg* and *wde* in germ line cells by FLP/FRT mediated mitotic recombination. Egg chambers with *egg* or *wde* germ line clones did develop up to stage 8 of oogenesis, but subsequently degenerated due to apoptosis. Because the ovary phenotype of homozygous mutant *egg* and *wde* females was more severe than the germ line clone phenotype of mutants in both genes, we conclude that *wde* and *egg* may also be required for proper development of somatic follicle cells.

It has been speculated that Egg may be dispensable for trimethylation of H3K9 at later stages of oogenesis because this function could be taken over by Su(var)3–9 [38]. However, this hypothesis is not consistent with the different localization of the Wde/Egg complex and Su(var)3–9 on salivary gland polytene chromosomes and with the different consequences of the respective mutations on H3K9 methylation in pericentric heterochromatin and euchromatin, in particular on chromosome 4 [18],[35],[37]. Furthermore, mutations in *wde* and *egg* lead to apoptosis of germ line cells, which obviously cannot be rescued by the presence of Su(var)3–9 which is already expressed in the germ line at the time when apoptosis starts.

### SUMOylation of Egg may recruit additional chromatin modifiers

Modification by SUMOylation and binding to SUMO is a common hallmark of many chromatin regulators involved in transcriptional repression [32]. Both mAM/MCAF1 and SETDB1 can bind SUMO and it has been suggested that this property is required for the recruitment of these proteins to promoters bound by transcriptional repressors such as KAP1, Sp3 and MBD1 [33],[34],[47],[48]. Our finding that Egg is itself modified by SUMOylation suggests that binding of additional chromatin modifiers to SUMOylated Egg may contribute to the efficient assembly of higher order chromatin repression complexes at specific euchromatic sites.

## Materials and Methods

### Fly stocks and genetics

The following stocks were used in this study: P{EP}EP2024 (Szeged *Drosophila* Stock Center), P{XP}CG12340^d06198^, P{XP}d03942, P{XP}d01917 (Exelixis collection at Harvard), P{EPgy2}CG12340^EY00884^ (#15045), Df(2R)27 (#8109), daughterless-GAL4 (#5460), engrailed GAL4 (#6356), tubulin-GAL4 (#5138), mat67-GAL4 (#7062), P{w+FRTG13}GFP (#5826), P{neoFRT40A} P{w^+^FRTG13} (#8217), y w hsFlp; Sco/CyO (#1929) (Bloomington *Drosophila* stock center, stock # given in parentheses), *egg* ^1473^ [36]. A chromosome doubly mutant for *wde^06198^* and *egg^1473^* was generated by meiotic recombination. The *wde^TD63^* null allele was generated by FLP/FRT mediated recombination in trans of the P{XP}d03942 and P{XP}d01917 P element insertions [49]. Expression of UASP-GFP-Wde, UASP-Egg-RFP, UASP-POF-RFP and of endogenous Wde from the P insertion P{EP}EP2024 [50] in transgenic flies was done with the UAS-GAL4 system [51]. Germ line clones for *wde* and *egg* were generated as described using a heat shock promoter driven flippase on the X-chromosome [43]. Transgenic fly lines for the constructs pUASP-GFP-Wde, pUASP-Egg-RFP and pUASP-POF-RFP were generated as described [52]. To obtain homozygous *wde* mutant adults, living embryos lacking GFP fluorescence derived from the CyO[twi::GFP] balancer chromosome were separated from their GFP positive siblings under a fluorescence stereo microscope and raised in separate vials.

### Antibodies and immunohistochemistry

Antibodies against Wde were generated by immunizing two rabbits with the following peptides: DKPKKISDRERNPGS (aa 70–84) and RSENTPPPASRLRYSH (aa 1286–1301). Final bleeds of both rabbits were pooled and affinity purified against the peptide corresponding to aa 70–84 (Eurogentec, Seraing, Belgium). For immunohistochemical stainings of embryos, ovaries and salivary gland polytene chromosomes the following antibodies were used: rabbit anti Wde, affinity purified (see above), 1∶5000 (1∶500 for polytene chromosomes); rabbit anti POF, 1∶1000 (1∶400 for polytene chromosomes) [41]; rabbit anti activated Drice, 1∶2500 [44]; rabbit anti SUMO, 1∶500 [53]; rabbit anti H3K9me2, 1∶500 (Upstate 07–441); rabbit anti H3K9me3, 1∶500 (Upstate 07–442); rabbit anti GFP, 1∶1000 (Abcam ab65556); mouse anti GFP 3E6, 1∶1000 (Invitrogen); mouse anti GFP, 1∶1000 (Roche 11814460001); mouse anti HP1 C1A9, 1∶25 (DSHB); mouse anti Orb 4H8, 1∶25 (DSHB). Secondary antibodies conjugated to Cy2, Cy3 (Jackson Laboratories) and Alexa 647 (Invitrogen) were used at 1∶400. DNA was stained with DAPI. Ovaries were fixed in 4% formaldehyde/PBS and stained according to standard procedures. Embryos were fixed and stained as described [54]. Polytene chromosomes were prepared and stained as described [55]. Images were taken on a Zeiss LSM 510 Meta confocal microscope and processed using Adobe Photoshop.

### Western blots and immunoprecipitation

Lysates of S2 cells were prepared in TNT buffer (150 mM NaCl; 50 mM Tris-Cl pH 8,0; 1% Triton X-100) supplemented with protease inhibitors (Roche). Western blots and coimmunoprecipitations were done as described [56]. For Western blots, the following antibodies were used: rabbit anti Wde, affinity purified, 1∶1000; rabbit anti POF, 1∶3000 [41]; rabbit anti Sumo, 1∶5000 [53]; rabbit anti actin A2066, 1∶1000 (SIGMA); mouse anti GFP, 1∶1000 (Roche 11814460001); mouse anti HA 12CA5, 1∶1000 (Roche).

## Supporting Information

Figure S1 The anti Wde antibody specifically detects endogenous Wde and overexpressed GFP-Wde. (A,B) A wild type embryo at stage 16 was stained for DNA (DAPI, turquoise, (A)) and Wde (red, (B)). Note the intense staining of Wde in primordial germ cells (arrows). (C,D) Wde staining is strongly reduced in a *wde^TD63^* homozygous mutant embryo at the same stage. Note that residual maternal Wde can be detected in the primordial germ cells ((D), arrows). Homozygous mutant *wde^TD63^* embryos were identified by absence of lacZ staining derived from the Cyo[ftz::lacZ] balancer chromosome (lac Z staining not shown). (E–G) Overexpressed full length GFP-Wde is detected by the anti Wde antibody. pUASP-GFP-Wde was overexpressed under control of the engrailed-GAL4 driver line, which is expressed in segmentally repeated stripes in the epidermis. The GFP fluorescence (E) matches precisely the staining with the anti Wde antibody (F,G). (H,I) Endogenous Wde was overexpressed under control of engrailed GAL4 using the P{EP}EP2024 insertion line (I) in which the EP element is inserted 40 bp upstream of the transcription start site of the wde locus. The overexpressed Wde was detected by the anti Wde antibody (H). (J–M) In the embryonic ectoderm, Wde (red, (K–M)) is nuclear in interphase cells and shows partial colocalization with the DNA dye YoYo-1 (green, (J,L,M)). In mitotic neuroblasts (asterisks), Wde is cytoplasmic and does not colocalize with DNA (K–M). Neuroblasts were marked by expression of Miranda (blue, (M)). Scale bars in (A) and (E) = 100 µm, Scale bar in (J) = 10 µm. Anterior is to the left in all panels.
(7.66 MB TIF)

Figure S2 *wde* and *egg* homozygous mutant females possess only rudimentary ovaries. Whole ovaries of 2 day old wild type (A,B), *wde^TD63^* (C,D), and *egg^1473^* homozygous mutant females (E,F) were stained with DAPI. Whereas wild type ovaries contain approximately 16 ovarioles each with egg chambers at different developmental stages (A,B), both *wde* and *egg* mutant ovaries are tiny and do not contain any egg chambers that have separated from the germarium (C–F). Boxes indicate regions shown at higher magnification in the right panels. Scale bars = 200 µm.
(1.89 MB TIF)

Figure S3 Wde is dispensable for oocyte determination. (A–J) GFP marked control germ line clones (A–E) and *wde^00884^* mutant germ line clones (F–J) were induced using the FLP/FRT technique. Ovaries were stained for DNA with DAPI and for Wde (C,H) and Orb (D,I) using specific antibodies. Germ line clones are marked by the absence of GFP fluorescence ((B,G), dotted circles). While control germ line clones show nuclear Wde staining in nurse cells and in the oocyte (C), *wde* mutant germ line cells lack nuclear Wde staining (H). Both in control clones and in *wde* mutant clones, oocyte determination appears normal, because staining for Orb is restricted to a single cell at the posterior pole of each egg chamber (D,I). Scale bars = 50 µm.
(2.46 MB TIF)

Text S1 Supplemental methods.
(0.03 MB DOC)
