# Supplementary material for: Windei, the Drosophila Homolog of mAM/MCAF1, Is an Essential Cofactor of the H3K9 Methyl Transferase dSETDB1/Eggless in Germ Line Development
Source: PLoS Genet. 2009 Sep 11;5(9):e1000644. doi: 10.1371/journal.pgen.1000644 (PMC2730569; doi:10.1371/journal.pgen.1000644)
Supplement: Text S1 — Supplemental methods. (0.03 MB DOC) [file pgen.1000644.s004.doc]

**Supplemental Material**

**Supplemental Methods**

Plasmid construction for expression of Wde, Egg and POF in S2 cells and transgenic flies

Full length and deletion constructs of Wde, Egg and POF were generated by amplification of the respective DNA fragments by PCR, using the full length EST clone GH06075 (Wde) or cDNA amplified from poly A+ RNA (Egg, POF) as templates. For PCR, the following primer combinations were used (always in 5’-3’ orientation; the first sequence is the forward primer, the second sequence the reverse primer):

Wde-1: CACCATGATGGGAGTAAACCAG, CTACGACCAGGTCTTGGGAACAC;

Wde-3: CACCATGGAGCTGGTGCTGCAGAA, GAAATGAGAGTTCTTAGCCTCCA;

Wde-4: caccatgatgggagtaaaccag, cagcaccagctcctccaggt;

Wde-5: caccatgtctcatttcaccgctcc, cgaccaggtcttgggaacac;

Egg-1: CACCATGTCTGGGCAGCCAACAGC, GAGCAGACGAAGGCGGCAAT;

Egg-2: CACCATGTCTGGGCAGCCAACAGC, TTGGGACACGCAGCTGCTGTG;

Egg-3: CACCATGTACAAGTGCCACGAGTG, GAGCAGACGAAGGCGGCAAT;

Egg-4: CACCATGTCTGGGCAGCCAACAGC, GGCAATGTGCTTGGCCGTCA;

Egg-5: CACCATGAGTCGACCAGCAGAAGA, TGACCAGGACAGCTGAGCCG;

Egg-6: CACCATGTCTGGGCAGCCAACAGC, GTATTCTTCTGCTGGTCGACT;

Egg-7: CACCATGGAAGATGAACTCGATGATGCC, GGCAATGTGCTTGGCCGTCA;

POF: CACCATGGATTCAAAACGCGCGGC, AGGATCAGGATCGCGTTCCAG

PCR products were cloned into pENTR (Invitrogen) according to the manufacturers instructions. Epitope tagged expression constructs were generated by recombination of the respective inserts from the pENTR clones into different destination vectors encoding C- or N-terminal GFP or HA epitope tags using the Gateway technology (Invitrogen) and the *Drosophila* Gateway vector collection (*Drosophila* Genomics Resource Center).

**Supplemental Figure Legends**

Figure S1. The anti Wde antibody specifically detects endogenous Wde and overexpressed GFP-Wde. (A, B) A wild type embryo at stage 16 was stained for DNA (DAPI, turquoise, A) and Wde (red, B). Note the intense staining of Wde in primordial germ cells (arrows). (C, D) Wde staining is strongly reduced in a *wde^TD63^* homozygous mutant embryo at the same stage. Note that residual maternal Wde can be detected in the primordial germ cells (D, arrows). Homozygous mutant *wde^TD63^* embryos were identified by absence of lacZ staining derived from the Cyo[ftz::lacZ] balancer chromosome (lac Z staining not shown). (E – G) Overexpressed full length GFP-Wde is detected by the anti Wde antibody. pUASP-GFP-Wde was overexpressed under control of the engrailed-GAL4 driver line, which is expressed in segmentally repeated stripes in the epidermis. The GFP fluorescence (E) matches precisely the staining with the anti Wde antibody (F, G). (H, I) Endogenous Wde was overexpressed under control of engrailed GAL4 using the P{EP}EP2024 insertion line (I) in which the EP element is inserted 40 bp upstream of the transcription start site of the *wde* locus. The overexpressed Wde was detected by the anti Wde antibody (H). (J – M) In the embryonic ectoderm, Wde (red, K - M) is nuclear in interphase cells and shows partial colocalization with the DNA dye YoYo-1 (green, J, L, M). In mitotic neuroblasts (asterisks), Wde is cytoplasmic and does not colocalize with DNA (K – M). Neuroblasts were marked by expression of Miranda (blue, M). Scale bars in (A) and (E) = 100 µm, Scale bar in J = 10 µm. Anterior is to the left in all panels.

Figure S2. *wde* and *egg* homozygous mutant females possess only rudimentary ovaries. Whole ovaries of 2 day old wild type (A, B), *wde^TD63^* (C, D) and *egg^1473^* homozygous mutant females (E, F) were stained with DAPI. Whereas wild type ovaries contain approximately 16 ovarioles each with egg chambers at different developmental stages (A, B), both *wde* and *egg* mutant ovaries are tiny and do not contain any egg chambers that have separated from the germarium (C – F). Boxes indicate regions shown at higher magnification in the right panels. Scale bars = 200 µm.

Figure S3. Wde is dispensable for oocyte determination. (A – J) GFP marked control germ line clones (A – E) and *wde^00884^* mutant germ line clones (F – J) were induced using the FLP/FRT technique. Ovaries were stained for DNA with DAPI and for Wde (C, H) and Orb (D, I) using specific antibodies. Germ line clones are marked by the absence of GFP fluorescence (B, G, dotted circles). While control germ line clones show nuclear Wde staining in nurse cells and in the oocyte (C), *wde* mutant germ line cells lack nuclear Wde staining (H). Both in control clones and in *wde* mutant clones, oocyte determination appears normal, because staining for Orb is restricted to a single cell at the posterior pole of each egg chamber (D, I).
